# Tuberculosis as Public Health Problem in the Slovak Republic

**Published:** 2017-06

**Authors:** Martin SAMOHYL, Ivan SOLOVIC, Jana SVECOVA, Roman RAMS, Katarina HIROSOVA, Jana JURKOVICOVA

**Affiliations:** 1. Institute of Hygiene, Faculty of Medicine, Comenius University, Bratislava, Slovakia; 2. Institute of Tuberculosis, Lung Diseases and Thoracic Surgery, Vysne Hagy, Slovakia; 3. Catholic University, Ruzomberok, Slovakia; 4. National Health Information Center, Bratislava, Slovakia; 5. Institute of Public Health, Jessenius Medical Faculty Comenius University, Martin, Slovakia

## Dear Editor-in-Chief

Tuberculosis (TB) is a leading cause of global morbidity and mortality and still global public health problem (1, 2). The worldwide annual incidence of the active TB forms is more than 8 million, with more than 90% of deaths in developing countries; therefore, tuberculosis remains one of the most important global threats in public health (3). Tuberculosis is the most common cause of death of all treatable infectious diseases (4).

We conducted a descriptive analysis of data reported in the National Tuberculosis Registry authorized by the National Health Information Centre. Patient-specific data (i.e., demographic, clinical, epidemiologic, and laboratory information) were collected by using a standardized TB case notification forms.

**Fig. 1:**
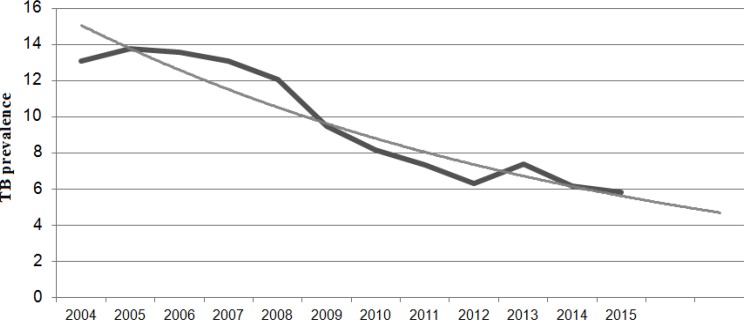
The TB prevalence in Slovakia (trend analysis) 2004 to 2015

The prediction of the TB incidence decrease in Slovakia (Fig. 1), relevant in the 80s of the 20th century, is now being fulfilled. The highest TB reported cases in Slovakia was in 1960 when there were 7817 registered TB cases, and since then it has a declining tendency. In the second half of the 80s, the decline was even more significant. In 2002, there were 1053 reported cases of newly diagnosed TB and in 2003 the number of newly diagnosed cases was less than one thousand for the first time. TB decreased by >25% in all age groups over the period in Slovakia. The highest TB prevalence was in the Presov region (PO, 13.07/100000 population). In 2015, 317 TB cases were reported (prevalence of 5.85/100000 population), of 28 were relapses. In 2014, no cases of co-infection of TB and HIV were reported. In total, 222 TB cases were verified (66.1%) – by bacteriological, histological and other methods. In 2014, the Slovak Republic achieved the highest rate of successful treatment (85%) of newly diagnosed and microscopically confirmed TB cases in European countries. In 2015, a higher TB incidence was in men in age groups 25–79 yr than in women, with the highest incidence in the 55–59 age group (23 cases). In women, there was a higher TB incidence in the 0–19 age group, with the highest TB incidence in the 0–4 age group (22 cases). The Roma population had 100% rate on the TB prevalence in the 15–19 yr age group. The TB prevalence in childhood has been increasing since 2010. In 2015, TB was diagnosed in 67 children; of 53 cases were Roma children. The highest TB prevalence in children was in the Presov and Banska Bystrica regions. The Trnava, Zilina, and Bratislava regions have not reported any TB cases in children. The Slovak Republic achieved the highest rate of successful TB treatment (85%) in Europe. Over the years 2003–2015, the highest TB prevalence in Slovak prisons was in the year 2007 (34 cases). The most deaths in TB patients (with no TB cause of death) in Slovakia was in 2007 (47 cases) and the less in 2014 (13 cases). The most common methods of TB detection in patients included visiting outpatient departments for problems (221 cases), TB contacts examinations (55 cases), preventive medical checks (10 cases), checking the registered TB patients (30 cases) and autopsy (1 case).

Medical education relating to TB is continuing and up to date. Conferences, meetings, workshops and case studies are jointly organized on an almost monthly basis by the TB center, the Slovak Society of Respiratory Diseases and other academic institutions. This is reflected by the teaming of GPs with assistants from the Roma communities. The GPs ensure early referral and treatment follow-up, helped by the Roma assistants through their community links with hard-to-reach populations. This facilitates not only adherence to and follow-up after treatment but also education about the prevention of TB and investigation of contacts of people with TB.

In Jan 2012, the government of Slovak Republic decided to stop obligatory Bacille Calmette-Guerin (BCG) vaccinations in view of the low incidence and notification of TB in the country. Since then, there has been a major debate for and against this decision. In 2013, Regional Public Health with a seat in Poprad ordered, based on the epidemiological situation, compulsory vaccination in newborn’s aged from four days to six weeks in three municipalities (Vyborna, Krizova Ves, Hranovnica).

In Slovakia, the incidence of TB decreased in recent years, but it is necessary to pay attention to ill individuals, especially in high-risk populations – among the elderly, the unemployed, homeless, alcoholics and in the Roma ethnic group. In the Slovak Republic, the TB incidence has a decreasing tendency in recent years, but more attention should be paid to finding and treating the patients, particularly in high-risk population groups.
